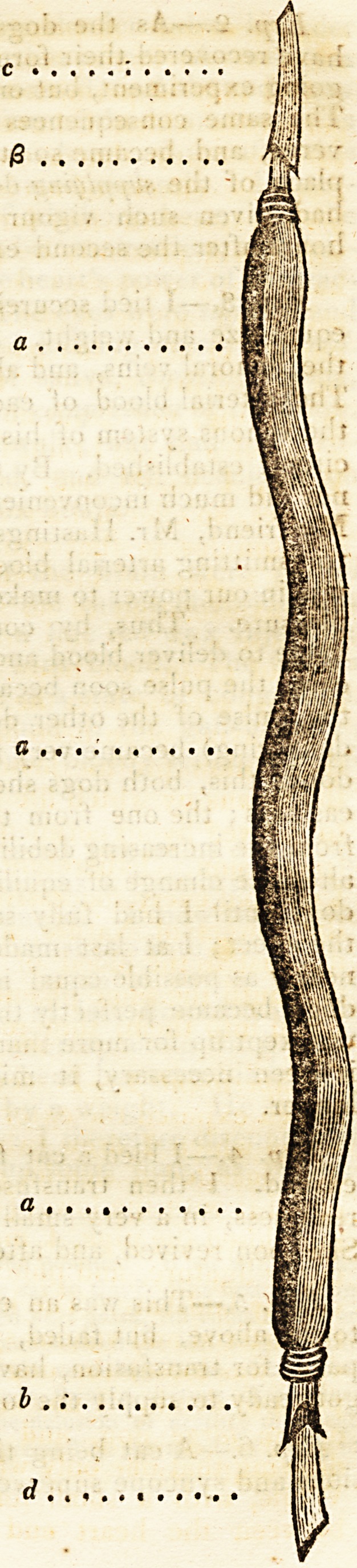# The Observer. No. I

**Published:** 1817-04

**Authors:** 


### Medico-Chirurgical

### JOURNAL AND REVIEW.

VOL. III.] APRIL, 1817. [NO. 16.

### PART I.

### ORIGINAL COMMUNICATIONS.

### THE OBSERVER. No. I.

Quærere verum.

### ART. 1. GOUT.

THE *Eau Medicinale* of Husson had its day; and then fell—not merely into neglect, but execration! The fate of Dr. Kinglake's *Medicinal Water* was little better. A near cut to health proved, in numerous instances, but a near cut to the grave; and when the able treatises of Drs. Parry and Scudamore placed the Gout in its true light, the prophetic words of Cullen, " patience and flannel," came forcibly to the recollection of the observant members of the profession.

Expellas naturam furca tamen usque recurrit!

The attentive and enlightened physiologist, perceiving that Gout was almost invariably preceded, accompanied, and followed by constitutional symptoms, became convinced that it was not to be combated by *local* means without hazard to the general health. This observation was corroborated by the many dangerous and even fatal affections which supervened on the sudden *spontaneous* retrocession of Gout; numerous cases of which almost every observant practitioner must have met with in his own sphere of action. I shall only quote one short passage in support of this, from the first physician of the age we live in.

"On the other hand, various diseases of the head, as head-ache, vertigo, depression of spirits, mania, epilepsy, and apoplexy, in many instances *immediately* or soon succeed the recession of inflammatory gout from the extremities." *Parry's Elements of Pathology*, p. 382.—It was not, therefore, without some degree of surprise, if not scepticism, that I lately heard of a "new, simple, and *expeditious* mode of curing Gout," by Dr. Balfour, of Edindurgh; and as I have had an opportunity of seeing some of the "subitæ mortes et intestata senectus" which resulted from other *new and expeditious* modes of curing this obstinate disease, I perused Dr. B's "Observations and Cases" with no trifling interest.*

Dr. Balfour remarks, that he sees nothing in the nature of things why percussion and compression should not be as beneficial in gout as in rheumatism. But surely this is not good logic. The two diseases are different in their nature; and what reasons, *a priori* have we then to conclude, that the same remedies should suit both. If it be said that gout and rheumatism are often blended, so are scrofula and syphilis; but who would argue from this, that mercury is as "beneficial in the one disease as in the other?" But to the cases. "Madame Rey was attacked, early one morning, with a *most violent* paroxysm of gout, in one of her great toes. She called her servant, and ordered her to *compress and beat the part*. This was done with moderate force, which excited the pain to a *most torturous pitch*. In a fit of despair, Madame Rey commanded the girl to use *all her force*, which was attended with immediate and complete relief from pain." I ask Dr. Balfour, if he ever experienced a "*most violent*" attack of this disease in his own person? If not, was it ever his lot to give an unfortunate jolt or squeeze to the great toe of an arthritic patient or friend writhing under a "*most torturous*" paroxysm of gout? Let doctor or patient put these questions to themselves, and the exaggerations of the ultra-arthritic Madame Rey will excite a smile of incredulity.

God forbid that I should entertain or express the slightest doubt of the fidelity or authenticity of Dr. Balfour's

---

* Edinburgh Med. and Surg. Journal, October 1816, p. 432.

own cases. Yet the records of medicine, and every physicians own memory, furnish indubitable proofs of the fallacy which creeps in on every experiment that is made with a favourite, and much more a self-invented remedy. How often has the veil of prejudice concealed the most palpable truths from our eyes! How often has the jaundice of a preconceived opinion, like Circe's wand, transformed and distorted every object that came within the field of our mental vision!

When Dr. Balfour applied percussion and compression to chronic rheumatism, he did that which has been done time immemorial in the Eastern world, and which was put in practice in this country at least thirty years ago.* As these circumstances were doubtless unknown to him, he has all the credit of a discovery. But when he extends these mechanical means to acute rheumatism and to gout, he treads on *tender ground,* and I stake my prophetic character on the eventual rejection of this plan from the practice of the enlightened orders of the profession, if indeed they should ever take it up.

There is something equivocal in the cases themselves. The *first* is far from being a clear and well-marked case of gout. Nothing is said of the patient's ever having had a *previous* attack, while the exciting causes, over-heated exertion and subsequent neglect, are those which more frequently induce transient rheumatism than gouty inflammation.

In the second case, the patient walked to the Doctor's house, and was merely lame from "pain in the soles and ankles." Percussion and a bandage, in this instance, effected an almost instantaneous cure. But on the 22d of the ensuing month, this gentleman was visited with a still more severe attack, which certainly had the appearance of gout, and was accompanied by "sickness, great uneasiness, and oppression." The mechanical process and a dose of opening medicine operated as before, like a charm, and in little more than twenty-four hours the arthritic patient was abroad!

The philosopher's reply to Crœsus should never be forgotten by the physician — "Mark the end." The publication of *cures* leaving a veil over *consequences* has been the cause of infinite evil in the medical world. On these two solitary cases, and Madame Rey's *ultra*-exaggeration, Dr.

---

* Vide Med. Chir. Journal, vol. iii. page 109.

Balfour has hastened to lay before the medical public "a new, simple, and expeditious mode of curing Gout."

"It is the general opinion (says Dr. B.), and it has been sanctioned by the first medical authorities, that *duration of inflammation* in the extremities, for a considerable time, is equally conducive to the health of the body and to the vigour of the mind. This *conceit*, for it deserves no better name, tends, more than all other considerations combined, to reconcile gouty patients to protracted sufferings." When an individual, from such materials as the above two cases, draws a conclusion, that the general opinion of the profession, sanctioned by the first medical authorities, is a mere *conceit*, it stamps that individual with the seal of presumption, so deep and so indelible, as to invalidate every antecedent and subsequent testimony from his pen. But Dr. Balfour has unfairly represented the general opinion on this subject. The first medical authorities do *not* attribute the salutary operation of a gouty paroxysm to the mere "duration of inflammation" in the extremities; but the enlightened and observant pathologist sees, that a *constitutional* indisposition, a gouty diathesis, or whatever other term may be applied, is safely expended, as it were, at a distance from vital parts, during the confinement; the anorexy, the reduction of plethora, the pain, and the irritation of a gouty paroxysm in the extremities. If this were not the case, how is it that various uneasy feelings, and disturbances of internal functions, almost invariably *precede* the paroxysm, while pleasant sensations and improved functions as regularly *follow* the attack? Have the two cases above mentioned, whose *after* history is unknown, obliterated from Dr. Balfour's memory the innumerable authentic instances of dangerous conversions of gout into visceral commotions and diseases? Can Dr. Balfour so far blind himself by a favourite hypothesis, as to suppose that a trifling difference in the local application [cold or compression] can materially affect the principle of conversion, in which consists the great danger of "new, simple, and expeditious modes of curing Gout." But I believe that several circumstances will conspire to circumscribe the danger of Dr. Balfour's practice. *First*, The noise which the waters of Husson and Kinglake made in the medical world, have, like the cataracts of the Nile, left a salutary *deafness* on the mental auditories of the Faculty, which may not speedily be effaced by any *change of peal* from a similar quarter. *Secondly*, I am convinced, that such is the degree of exquisite sensibility in arthritic inflammation, that no eloquence or authority would ever be able to introduce the practice of percussion and compression generally in that malady. *Thirdly,* I firmly believe, notwithstanding the cases of Madame Rey and Dr. Balfour, that the mechanical process, even should it be submitted to, is incapable of removing gouty inflammation, in the "new, simple, and expeditious" manner represented; and that the apparent relief of pain proceeds only from the cessation of previous percussive torture, in the same manner as we feel a temporary ease after holding a scalded or burnt surface for a few seconds near the fire. *Lastly,* I maintain, that, should my three first positions be falsified by the event, and Dr. Balfour's plan prove as instantaneous in the removal of Gout as the medicinal waters of Husson and Kinglake, the same immediate or remote evil consequences will ensue from the "new" as from the old *expeditious* modes of cure.

---

### ART. 2. HYDRENCEPHALUS.

THERE are few diseases, excepting perhaps obscure affections of the heart, that occasion more mistakes and false reasoning, both in practice and publications, than Hydrencephalus. In few instances has the want of precision in terms, or the misapplication of a name, produced greater confusion or greater contrariety of opinion. The inexperienced is alternately buoyed up with the hope, and cast down with the despair, of curing this malady. The fact appears to be, that Hydrencephalus is an unfortunate appellation, since it is made to include various and dissimilar diseases. Is it not the *last* stage, or consequence, in many cases, of infantile remittent fever, of phrenitis, of dentition, of worms, of hepatic congestion, of various intestinal irritations, &c.? And does it ever exist (excepting when congenital) without being preceded by some of these complaints? This last question is of some consequence. Since Whytt and Fothergill, who supported the idea of watery effusion taking place in the brain without previous excitement, as in the case of ordinary dropsy in other parts, Quin, Cheyne, Curry, Yeats, and Clarke, by their writings, have rendered the opinion general, that effusion is the consequence or cure of the preceding excitement, whether that excitement originated in the brain itself, or was determined by sympathy with a more distant organ. But what will be said to the experiments of Dr. Seeds,*

* Med. Ch. Journal, vol. i.

which prove that, in every instance of animals *bled to death*, there is an overwhelming effusion of water in the ventricles and between the coverings of the brain? These experiments go far to prove, that Hydrencephalus may result from *inanition*, as well as from the various other diseases and states of the system alluded to. This being the case then, can we, with propriety, apply the term Hydrencephalus to any particular disease or series of symptoms? Or rather, is it not the consequence, the effect, the ultimate stage, or, without meaning a pun on so serious a subject, the *caput mortuum* of any or every infantile disease? This seems to have been, in some degree, the impression on Dr. Clarke, who, in his work on the Diseases of Children, does not treat of Hydrencephalus under that name at all, but under the head of *Phrenitis.* In this respect he has probably been more happy than Dr. Yeats, from whose admirable symptomatology, and keen penetration, much light was to have been expected on the subject. Let any one consult Dr. Cheyne's various forms and stages of Hydrencephalus, and he will soon be convinced, that that observant author describes a variety of infantile diseases that lead occasionally to the bourne of Hydrocephalus, whence *nulla vestigia retrorsum!* It is from this error that we every day see published successful cures of Hydrencephalus, where no particle of the disease existed. I shall quote the last that has met my eye.

Mr. Watson, of Stourport, relates the following Case of Hydrocephalus, "successfully treated," in the February Number of the London Medical Repository, p. 102.

S. Winnal, ætat. 7, was observed to pick her nose for ten days, and had irregular bowels. On the 5th of November, *she ate a quantity of chesnuts.* "She passed a restless night, talked in her sleep, could not eat in the morning, and was observed to *draw one leg after her.*" 2d day; no stool, skin hot, pain on pressure of the abdomen, screams out violently sometimes. Took an emetic, and afterwards a purgative of scammony and calomel. 3d day; the medicine had not operated properly. Scammony and calomel, and salts and senna, every two or three hours. Dover's powder at night. 5th day; lower extremities paralytic, as also the bladder and right arm; bowels copiously evacuated of *dark-coloured* slimy fæces; pain in the region of the *liver* on pressure; abdomen full. Six leeches to the hepatic region; afterwards a blister, and then three grains of calomel every second hour. 7th day; has taken 48 grains of calomel; stools more natural; bowels kept open by glysters. Her nights are now better, and her appetite returns. From this time she had a slow convalescence, and recovered.

Now I would ask Mr. Watson, on what grounds he denominates this disease Hydrocephalus? The only two symptoms that can at all appear to belong to Hydrocephalus, are the screaming and paralysis? But has Mr. Watson never observed these in various other infantile diseases? That *intestinal irritation*, aggravated by the chestnuts eaten, was the sole cause of that train of morbid phænomena which followed, will, I think, be acknowledged by every intelligent and experienced practitioner. That this *might not have* ultimately led to Hydrencephalus I will not take upon me to say; but I will venture to assert, that at no period of the disease had it any the slightest claim to the appellation of Hydrencephalus.

As all authors agree, that the *effusion*, or Hydrencephalus, properly so called, constitutes the *last* or most fatal stage of the disease, how can the appellation properly apply to the early stage, which is frequently cured before the effusion can take place? This reminds us of the American name for yellow fever—"*el prieto vomito*," or black vomit, which constitutes merely the *last* or fatal stage of the disease, and which, of course, does not exist in numerous cases of yellow fever that are cured. So, inflammation might, with equal propriety, be termed mortification or suppuration, because it frequently terminates in these last; or pneumonia might be denominated hydrothorax, because it sometimes produces or ends in effusion of water in the lungs or chest.

But this is not a mere matter of nosology or terminology; it bears strongly on points of practice of the utmost importance. Thus, if in yellow fever, phlegmonous inflammation, and pneumonia, it would be absurd, as well as improper, to take into our views of treatment black vomit, mortification, and thoracic effusion, excepting as terminations to be dreaded; so, in the various forms of infantile diseases, Hydrencephalus is a term that should be applied to none (till effusion takes place), though it may be dreaded as the worst conversion which the disease can take. Surely the treatment for *existing* Hydrencephalus is extremely improper when it is only impending; And if so, is it not preposterous to apply the same appellation to the original disease and the ultimate conversion? In phrenitis, for instance, which, according to the late and lamented Dr. Clarke, is so frequently converted into Hydrencephalus, the remedies in the one form would be death in the other! Hence then I conceive, that the term Hydrencephalus is not only improper in any set of symptoms where effusion in the brain has not actually taken place; but is liable to suggest wrong views, wrong remedies, and consequently to produce disastrous events.

VETUS.

( To be continued. )

---

Art. 3. *On the Transfusion of Blood in extreme Cases of Hæmorrhage.** By John Henry Leacock, M. D. of Barbadoes.

THE effects of Hæmorrhage may be either salutary or not. Numerous are the instances where Nature has relieved the system by a spontaneous flow of blood; and that artificial depletion has often effected the same purpose is well known. But there are other, and not a few cases, where profuse hæmorrhage, whether spontaneous, accidental, or by art, has been followed by a degree of exhaustion that has either immediately or remotely proved fatal. It is to these that my attention is now directed.

It may be asked, what is the quantity of blood necessary to constitute a *dangerous* hæmorrhage? The answer would be difficult and unsatisfactory. The age, the sex, the constitution, the state of health, and various other circumstances, as the order of bleeding vessels, their situation, the size of the wound, the rapidity of the flow of blood, the position of the body, &c. all influence the effects of hæmorrhage; so that the same quantity will, in different circumstances, produce different consequences. The first sign of the body being injured, or at least endangered, by hæmorrhage, is the cessation of the heart's action. As this organ cannot suddenly accommodate itself to the lessened volume of blood, it ceases to be excited, and syncope takes place. The blood forsakes the extreme vessels; the eyes are suffused with tears, and appear sunk; a death-like paleness usurps the previously rosy lips; and memory, volition, and sensation, are lost, in consequence of the brain no longer receiving its usual stimulus from the blood. This state is effected by Nature as a check to the hæmorrhage, and little is to be apprehended, provided the motion of the heart be not too long

---

* The substance of an Inaugural Dissertation defended last year in Edinburgh. Ed.

suspended. If it be, it cannot recover the power of motion, and death ensues.

If, during a hæmorrhage, syncope frequently takes place, remote evils of great magnitude may be the consequence, although immediate death may not ensue. The heart so slowly accommodates itself to the greatly diminished volume of blood in circulation, that the least excitation causes syncope. Voluntary motion becomes languid; the eyes lose their former brilliancy; the cheeks and lips assume a pallid hue; the pulse is quick, small, and fluttering. The breathing anxious and frequent; there is a ringing in the ears, and the whole nervous system becomes extremely irritable. The patient is alarmed by every noise, however gentle; and if any one touches the bed unawares, he starts up in terror. These are dangerous symptoms after a profuse hæmorrhage. The favourable signs are, a gradual diminution in the frequency of the pulse, and irritability of the nerves, which may be expected, if light food can be got to digest, and supply chyle to the blood-vessels.

Too often, however, a host of obstinate ailments follow, that are as puzzling to the physician as dangerous to the patient. From the deficiency of blood, and the irritability of the nerves, the usual secretions cease or become scanty, and all the functions are disordered. Dyspepsia and its Protean attendants next succeed. The pulse continues weak and quick, with distressing palpitation of the heart. The food lies undigested, and no blood is formed. Dropsical effusion is generally the next in train, which is sometimes local, but too often becomes general throughout all the cavities. These consequences of excessive hæmorrhage are sometimes successfully combated by the most assiduous and judicious attention; but much more frequently baffle the attempts of the most skilful physician.

Among the morbid effects of profuse evacuations of blood, we must not forget to mention subsequent *plethora*, to which, patients who are fortunate enough to recover from the above-mentioned evils, are particularly prone. Plethora is well known to be a preternatural distention of the vessels from an overabundant supply of chyle. Thus the lacteals which were excited to excessive action during the deficiency of blood and the progress of convalescence, require a considerable time and a well-regulated regimen, to wean them from their morbid activity. This plethora, unless cautiously checked, or speedily removed, will expose the patient to diseases scarcely less fatal than the other extreme; namely, to apoplexy, paralysis, troublesome hæmorrhages, &c.

If I may be permitted to reason from analogy, and experiments which I have made on animals, I would venture to affirm that a majority of those who have perished from the effects of accidental hæmorrhages, might have been restored to their former state of health, by the means which will presently be pointed out. When hæmorrhage has greatly weakened the frame, we in vain betake ourselves to active stimuli, whether applied to the nose, mouth, or any other part, for the heart can by no means be roused into action. The natural stimulus of the heart is wanting, nor can it contract itself sufficiently to furnish a stimulus to the brain, for want of vital fluid to act upon. What, I would ask, can promise, under such circumstances, so effectual a remedy as the application of a similar stimulus to that which has been withdrawn? What, in the science of medicine, can be more interesting, than the investigation of so simple a method of restoring life to those who are just crossing the threshold of eternity, and of giving the hope of a happy old age to those who are fast sinking into the arms of death?

It having often been my lot to contemplate the mischiefs that follow profuse losses of blood, and to try in vain all the means by which life is usually recruited, I determined to prove, by experiment, what are the consequences of transfusion. I therefore exhausted animals of their blood. I marked every successive symptom. I left the animals to the powers of Nature, and death invariably followed. I exhausted the blood of other animals to the same extent, and until the same phænomena ensued; but on transfusing blood into them, they recovered and throve.

*Experiment* 1.—I secured two dogs of different sizes. I then exposed the external jugular vein of the *lesser* dog, round which I drew two loose ligatures an inch apart. I next inserted a slender pipe through a small hole, and secured it with the lower [nearest the heart] ligature. Having closed the pipe, I made a puncture between it and the upper ligature, and punctured also the carotid artery, previously laid bare. The hæmorrhage was now profuse from the arterial and venous systems (the upper ligature on the vein was loose) the dog soon fainted, and seemed at the point of death. I immediately exposed the femoral artery of the *large* dog, and passed three ligatures round it, drawing that one only which was farthest from the heart. While, with a small forceps, I compressed the artery between the heart and the ligature nearest the heart,

I made a small incision for receiving a pipe between the middle ligature and hat farthest from the heart, which had been drawn tight. Keeping in readiness the upper ligature to stop hæmorrhage, I opened a communication between the two dogs thus:

I took a tube [*a a a*] about six inches long, made of an ox's ureter, and secured to each end of it a crow-quill; [*β b*] one of which [*β*] I introduced into the quill [*c*] which was attached to the jugular vein of the small dog; while that which was secured to the artery of the other animal [*d*] was introduced into the quill of the tube [*b*]. Every thing being now ready, I removed the forceps from the femoral artery of the large dog, when the blood passed rapidly into the vein of the other, through the artificial channel. In two minutes, the receiving dog that had been lying half dead, raised his head; the pulse, before imperceptible, began to be plainly felt; the eye that was previously dull and glassy, regained its former splendour; the respiration became easy and natural.—Soon afterwards he became impatient, and attempted to escape. I now put an end to the experiment: the dog ran about with ease, and appeared very little indisposed.

*Exp.* 2.—As the dogs, after a few days, appeared to have recovered their former strength, I repeated the foregoing experiment, but on the vessels of the opposite sides. The same consequences resulted: the *receiving* dog recovered, and became so strong that he afterwards took the place of the *supplying* dog; while the *latter*, whose blood had given such vigour to his comrade, died in twelve hours after the second experiment.

*Exp.* 3.—I tied securely on a bench two dogs of nearly equal size and weight. I fitted pipes of equal calibre to the femoral veins, and also to the femoral arteries in both. The arterial blood of each dog was thus transfused into the venous system of his companion, and a kind of double circuit established. By this plan they exchanged blood, nor did much inconvenience seem to result to either party. My friend, Mr. Hastings, holding in his hands the tubes transmitting arterial blood from each dog to his fellow, it was in our power to make the flow equal or unequal at our pleasure. Thus, by compressing a tube, one dog was made to deliver blood and receive none in return. In this case, the pulse soon became weak and intermittent; while the pulse of the other dog (which was receiving and not delivering) became very full and strong. While we were doing this, both dogs shewed the utmost anxiety and uneasiness; the one from the excess of stimulus, the other from the increasing debility. Having repeated this curious alternate change of equilibrium, four or five times in each dog, until I had fully satisfied myself and my friend of the effect; I at last made the flow through each tube as nearly as possible equal in force and velocity, when both dogs became perfectly tranquil. This double circulation was kept up for more than a quarter of an hour; and had it been necessary, it might have been continued much longer.

*Exp.* 4.—I bled a cat from the jugular vein till syncope ensued. I then transfused the blood of a dog I had in readiness, in a very small stream, into the vein of the cat. She soon revived, and afterwards recovered.

*Exp.* 5.—This was an experiment intended to be similar to the above, but failed, in consequence of the cat, prepared for transfusion, having died before the dog could be got ready to supply the loss of blood.

*Exp.* 6.—A cat being tied down, and bled till convulsions and syncope supervened, the arterial blood of a dog was transfused into her through a slender tube, in order that I might note the symptoms that arose. As soon as the blood reached the heart, she started, and shewed signs of returning animation. The action of the heart too, was distinctly felt to re-commence, by the hand. Soon after this she struggled, and the heart palpitated violently. I determined to examine the effects of plethora here. As the transfusion continued, the cat soon became restless, and *debility* advanced by hasty steps. The heart's power of contraction was evidently diminished. The eyes were *suffused* and slightly protruded, *glistening*, and looking *excessively red.* Next succeeded a copious flow of saliva, *vomiting*, coma, and apparently all the symptoms of *compression of the brain.* The cat, when let loose, tried to walk; but had not proceeded far, before she staggered, and fell headlong on the ground. She died in about six hours.

Dissection. The vessels of the brain were *gorged with black blood.* The vessels of the tunica choroides appeared as it were distended with a fine red wax injection. The tunica conjunctiva was red with blood. The viscera of the abdomen and thorax were in a state of congestion. A quantity of bloody bile was found in the gall-bladder; and in most of the cavities some bloody serum was effused.*

*Exp.* 7.—I laid bare and punctured two veins of a dog, the femoral and jugular. So copious a hæmorrhage ensued, that syncope was soon induced. I now transfused blood from the carotid artery of a sheep into the jugular vein of the dog. Upon this he began to revive and to struggle, though weakly. Blood at last flowed from the punctured vein. He attempted to creep along the ground, but always fell forward, with his head between his legs, as though he were pressed down by a weight. He evinced symptoms of a compressed brain: I therefore discontinued the transfusion. He now stood up some time, as if hesi-

* This experiment is worth ten thousand pounds! What will the advocates of Brunonian and Bulam debility say to this? The first symptom of *plethora* was *debility*, which advanced *pari passu*, till coma and every symptom of Bulam and tropical fever characterized the last stage of life. The suffused, protruded, glistening eye, the vomiting, coma, &c. corresponding with the *post mortem* appearances, form a picture of *plethora*, and read us a lesson of instruction on the delusion of *debility* that ought to be printed in letters of gold, and impressed on the minds of every individual in the profession.—Edit.

tating—then collecting all his strength, he rushed to the door, which he in vain attempted to open. He lived between twelve and fifteen hours, and did not suffer any great debility. After death, the blood was found quite fluid, and of a dark colour throughout the body.

*Exp.* 8.—I drew blood from a dog by the jugular vein; but before syncope supervened, I inserted a tube into the vein just below the orifice from which the blood flowed. Through this tube I transmitted blood from the carotid artery of a large sheep. The dog began immediately to struggle, and to bleed at the former wound, nor did any change appear in the colour of the blood, while the experiment lasted. I continued the experiment long, that, if possible, the dog might lose the whole of his blood. On this account, I endeavoured to regulate the flow of arterial blood according to the force with which the stream issued from the vein, and the manner in which the dog appeared to be affected.

No other dog seemed to suffer as much inconvenience from transfusion of blood as this. He could, indeed, stand and walk, but he was very weak. After thirty-eight hours he died.—On dissection, no adequate cause of death could be traced in any part of the body; nor any thing extraordinary, except the dark colour and fluid state of the blood.

*Exp.* 9.—On the 18th of February, I drew blood from the femoral artery of a young dog till syncope ensued: I then stopped the hæmorrhage by a ligature. Two days afterwards, on opening the same artery, very little blood flowed, for a large coagulum had nearly blocked up the whole passage. The next day I punctured the femoral artery of the opposite side. A profuse hæmorrhage was the consequence, and syncope again ensued.

On the 22d, when I saw that the dog had a little recruited, I punctured the humeral artery. The blood flowed freely, but it was paler than usual, and it coagulated immediately. The dog became excessively weak, wherefore I suppressed the hæmorrhage by a firm compress and bandage. These I removed two days afterwards, not thinking them longer necessary; but on returning in ten or twelve hours, I found that the poor dog had bled to such excess, that he was reduced to the lowest possible ebb. I again bound up the wound; but next day, on visiting him, I found his food untouched. He could no longer support himself on his legs, but immediately staggered and fell on being raised. His eyes were dull and obscure. His tongue, gums, and fauces blanched; and nothing but the most languid thrill could be perceived as a pulse. As it was evident that he must die in a few hours, I opened the external jugular, and transfused a few ounces of blood from a sheep. I then opened the vein of the dog a little above the former wound, and on the loss of ℥iß of blood the dog fainted away. I immediately transfused some blood into him from the carotid artery of a sheep, which instantly rendered the pulse more distinct; and the appearance of the dog was evidently changed for the better. He stood more firmly on his legs, though still languid. Six hours after this, the powers of motion returned gradually. Lastly, all those symptoms which, previously to transfusion, threatened death, entirely disappeared, and a true canine appetite ensued.

I think I need not adduce farther proofs of the safety, not to say utility of transfusion. A time, I hope, will come, when I shall be enabled to prosecute the investigation farther, and carry it to some useful end. Imperfect as the experiments are, I think they will remove all doubts from the liberal-minded reader respecting the safety of the process under consideration.

It appears clear, from a candid review of these experiments, that the blood of any animal, transfused into the vascular system of another animal of the same species, is sufficient to support life; and this not only in cases where the blood is partially transfused, as in the 1st, 2d, and 4th experiments, but even where almost the whole blood is changed, as in experiment 8. It is not, however, quite clear, that animals of *different species* will bear this transfusion to a great extent. For example, the 9th experiment shews that the blood of a sheep, an herbivorous animal, may support a dog, a carnivorous animal. But if the *whole blood* be drained from the receiving animal, the blood of the supplying animal appears not to answer the purpose. This is proved by experiments 7 and 8. It will never be necessary, however, that so much blood as I transfused in the 7th and 8th experiments, should be injected into a human subject: for, if the heart can be excited at all, a quantity of blood much less than has been lost, will be sufficient for that purpose. This is confirmed by my own experiments, and those of others.

As to the kind of animal from which the blood should be drawn, it seems of little consequence whether it be herbivorous or carnivorous. I would promise great advantage from either. As man, however, is omnivorous, it is probable that the blood of an omnivorous animal most resembles his; and therefore, that it would be most proper to supply its place. I have never analyzed the blood of different animals, but I am confident that there is a difference between the blood of different kinds, though this is denied by some. The phænomena which I observed in experiments 7 and 8, have strengthened my suspicions on this point.

How far transfusion may be useful in diseases connected with deficiency of blood, is highly worthy of investigation. I am induced to think most favourably of it. If, as is every day seen, the loss of blood can cure diseases arising from a redundancy of that fluid, what is there repugnant to the idea of trying to cure diseases arising from an opposite cause by an opposite remedy, to wit, by transfusion? The consequences of hæmorrhages, where the functions are not dangerously affected, do not, of course, require transfusion, since other remedies will suffice. But when the danger is imminent, and common means are ineffectual, as when a parturient woman trembles on the brink of the grave, from uterine hæmorrhage; or when a soldier is at the point of death from loss of blood, what reason can be alledged for not having recourse to this last hope, and for not attempting to recruit the exhausted frame, and turn the ebbing tide of life?*

* Would it not be better to transfuse the blood immediately from the vein of a healthy byestander. The blood would flow with ease from the vein of one person into that of another, by the apparatus above represented, by mere insertion into the orifices, keeping a bandage *above* the orifice of the supplying arm.—Edit.

## Figures and Tables

**Figure f1:**